# Is Independence Necessary for a Discontinuous Phase Transition within the *q*-Voter Model?

**DOI:** 10.3390/e21050521

**Published:** 2019-05-23

**Authors:** Angelika Abramiuk, Jakub Pawłowski, Katarzyna Sznajd-Weron

**Affiliations:** 1 Department of Applied Mathematics, Faculty of Pure and Applied Mathematics, Wrocław University of Science and Technology, 50-370 Wrocław, Poland; 2 Department of Theoretical Physics, Faculty of Fundamental Problems of Technology, Wrocław University of Science and Technology, 50-370 Wrocław, Poland

**Keywords:** opinion dynamics, voter model, phase transitions, Landau theory

## Abstract

We ask a question about the possibility of a discontinuous phase transition and the related social hysteresis within the *q*-voter model with anticonformity. Previously, it was claimed that within the *q*-voter model the social hysteresis can emerge only because of an independent behavior, and for the model with anticonformity only continuous phase transitions are possible. However, this claim was derived from the model, in which the size of the influence group needed for the conformity was the same as the size of the group needed for the anticonformity. Here, we abandon this assumption on the equality of two types of social response and introduce the generalized model, in which the size of the influence group needed for the conformity $q_{c}$ and the size of the influence group needed for the anticonformity $q_{a}$ are independent variables and in general $q_{c}\neq q_{a}$. We investigate the model on the complete graph, similarly as it was done for the original *q*-voter model with anticonformity, and we show that such a generalized model displays both types of phase transitions depending on parameters $q_{c}$ and $q_{a}$.

## 1. Introduction

Recently there has been an increased interest in discontinuous phase transitions in models of opinion dynamics [1,2,3,4,5,6]. The main reason for this interest may be the observation of the social hysteresis, which appears only in the case of discontinuous phase transitions. A hysteresis, i.e., dependence of the state on the previous states, was observed empirically in animal [7,8,9] as well as in human societies [10,11,12]. Moreover, it has been claimed for years that in social sciences, the hysteresis is typical rather than exceptional [10].

However, it has occurred that discontinuous phase transitions are not that typical in models of opinion dynamics, especially if we consider models belonging to the class of the binary-state dynamics [13,14] such as the voter model [15], the majority-vote model [15,16,17], the Galam model [18], the Sznajd model [19], the threshold model [6,20], the *q*-voter model [21] or the threshold *q*-voter model [22,23]. This is not very surprising, taking into account claims that the nonequilibrium spin systems with up-down symmetry fall in the universality class of the equilibrium Ising model, at least on regular lattices [1,17,24]. On the other hand, the above claim does not always have to be right. For example, it has occurred that the generalized voter and directed percolation universality classes are observed in models with two symmetric absorbing states [23,25,26]. These models can display continuous or discontinuous phase transitions, depending on the model parameters. One would therefore suspect that absorbing states are responsible for discontinuous phase transitions. However, the transition changes from continuous to discontinuous also within binary-opinion models without absorbing states [3,6,22,27], which will be discussed below. Moreover, recently the switch from a continuous to a discontinuous phase transition via a transcritical bifurcation was observed in a model of social contagion [28]. Yet, there are many opinion models with up-down symmetry that display only continuous phase transitions.

For example, within the original majority-vote model only continuous phase transitions are observed, even in the presence of an additional noise [1,5]. It was only the inertia, introduced on the microscopic level into the majority-vote model, that has changed the type of the phase transition from a continuous to a discontinuous one [2,4]. This result, however, might be criticized by the classicism of *‘You only get out what you put into it’*, because hysteresis is just an inertia at the macroscopic level. On the other hand, the assumption of the inertia on the microscopic scale, which means that a probability of flipping a spin depends not only on the states of its neighbors, but also on its own state, is very intuitive from the psychological point of view. Therefore, the inertia on the microscopic scale maybe indeed the realistic explanation of the hysteresis in the social systems. Nevertheless, one can also ask about the possibility of hysteresis as an emergent property in the system of memoryless agents and such a question has been asked within the *q*-voter model.

It has occurred that within several versions of the *q*-voter model, similarly as in the majority-vote model without inertia, only continuous phase transitions was observed, including the original *q*-voter model [29], the *q*-voter model with anticonformity [30], the *q*-voter model with zealotry [31,32,33] or the threshold *q*-voter model with anticonformity [22]. On the other hand, discontinuous phase transitions has been reported for the *q*-voter model with independence for q>5 [30], for the threshold *q*-voter model with independence [22], for the nonlinear noisy voter model [34], as well as for threshold *q*-voter model with the stochasticity that acts in the lack of majority [23]. The existence of a discontinuous phase transition was one of the reasons for which the *q*-voter model with independence has gained more attention than the *q*-voter model with anticonformity and has been investigated on various monoplex [3], as well as duplex networks [35].

Similarly, as for the *q*-voter model, also for the symmetrical threshold model (which can be interpreted as the super-majority-vote model) independence is needed for a discontinuous phase transitions and for the model with anticonformity only continuous phase transitions are possible [6].

Taking into account all these results, we ask the question whether independence is actually necessary for a discontinuous phase transition within the *q*-voter model or maybe anticonformity is sufficient if we abandon unjustified assumptions introduced in [30]. It has been assumed that the size of the unanimous group of influence needed for conformity is the same as the size of the unanimous group of influence needed for anticonformity. This assumption is not confirmed by any social experiment, but it made the model simpler in the sense of the number of parameters. Abandoning the assumption increases the number of parameters from two to three, but it is still not much in comparison to a very extensive model proposed in [36]. Therefore, on contrary to [36] we are able to systematically examine the model and find analytical formula not only for the critical point, below which an up-down symmetry is broken, but also to derive an analytical formula for the tricritical point, which proofs the possibility of the discontinuous phase transition for the *q*-voter model with anticonformity.

## 2. Model

We consider a system of *N* individuals that are tied to the nodes of an arbitrary graph. Each node of a graph is occupied by exactly one individual (wording “individual”, “agent”, “voter” and “spin” will be used interchangeably). Each of them can be in one of two states, described by the dynamical binary variable $S_{i}\left(t\right)=+1\left(↑\right)$ or $S_{i}\left(t\right)=-1\left(↓\right),\;i=1,\ldots ,N$ that represents an opinion on a given subject (yes/no, agree/disagree, etc.) at given time *t*. At each elementary update, a single node *i* is randomly chosen from the entire system. In a given update a voter at node *i* behaves like anticonformist with probability *p*, whereas with complementary probability 1−p it behaves like conformist. In the first case (anticonformity) a voter is influenced by the group of size $q_{a}$ and it rebels against the group. This means that $q_{a}$ agents are randomly chosen without repetitions from $k_{i}$ neighbors of a voter at site *i* to form a group of influence. If this group is unanimous, i.e., if all $q_{a}$ agents are in the same state then a voter at site *i* takes the opposite position to this group. If $q_{a}>k_{i}$ then an efficient group of influence cannot be formed, and the state of the system does not change. In the second case (conformity) a voter is influenced by the group of size $q_{c}$ and it adapts to the group. This means that $q_{c}$ agents are randomly chosen without repetitions from $k_{i}$ neighbors of a voter at site *i* to form a group of influence. If all $q_{c}$ agents that form a group of influence are in the same state then a voter at site *i* takes the same state as individuals in the group. If $q_{c}>k_{i}$ then an efficient group of influence cannot be formed, and nothing happens. In result only the following changes are possible:

(1) $$\begin{array}{ccc} \underset{q_{a}}{\underset{︸}{↑↑\ldots ↑}}⇑ & \overset{p}{⟶} & \underset{q_{a}}{\underset{︸}{↑↑\ldots ↑}}⇓\\ \underset{q_{a}}{\underset{︸}{↓↓\ldots ↓}}⇓ & \overset{p}{⟶} & \underset{q_{a}}{\underset{︸}{↓↓\ldots ↓}}⇑\\ \underset{q_{c}}{\underset{︸}{↑↑\ldots ↑}}⇓ & \overset{1-p}{⟶} & \underset{q_{c}}{\underset{︸}{↑↑\ldots ↑}}⇑\\ \underset{q_{c}}{\underset{︸}{↓↓\ldots ↓}}⇑ & \overset{1-p}{⟶} & \underset{q_{c}}{\underset{︸}{↓↓\ldots ↓}}⇓, \end{array}$$

where ⇑/⇓ denotes a state of a voter at node *i* and ↑/↓ denotes a state of an agent in the group of influence. For $q_{a}=q_{c}=q$ the model reduces to the original *q*-voter model with anticonformity [30].

In this paper, we will consider the model on a complete graph for two reasons. First, the original *q*-voter model with anticonformity has been investigated exclusively on a complete graph, so this structure is the best for the comparison with the previous results. Secondly, for this structure analytical calculations are particularly simple, and it is possible to derive exact analytical formula for the critical point below which an up-down (yes-no) symmetry is broken, as well as the tricritical point above which the phase transition becomes discontinuous.

Before we proceed to the results, let us make one comment regarding the choice of the group of influence. Originally repetitions in choosing neighbors were allowed to make the model universal, i.e., unambiguously defined for any type of graph and for any value of *q* [21]. Later on, the model without repetitions was introduced [3,30]. In a case of a complete graph two approaches give the same result. However, in general one must decide if repetitions should be allowed or not. Each of these two choices can be interpreted differently from the social point of view. For the model with repetitions one possible interpretation is the following: the change of an individual’s opinion is induced by the consecutive *q* meetings with people who can influence her/him, and we take into account that she/he can meet several times with the same person. On the other hand, for the model without repetitions a natural interpretation is the following: the change of opinion takes place under the influence of a unanimous group on which one is exposed at a given moment. Only the second possibility has been confirmed by social experiments [37] and thus we formulated the version without repetitions.

## 3. Results

In the case of a complete graph a state of the system is fully defined by a single aggregated quantity, such as an average concentration of agents with positive opinions:

(2) $$c\left(t\right)=\frac{N_{↑}\left(t\right)}{N},$$

where $N_{↑}\left(t\right)$ denotes the number of agents in the state ↑ at time *t*. Alternatively, we could choose an average opinion (magnetization), which is a natural order parameter [38]:

(3) $$m\left(t\right)=\frac{1}{N}\sum_{i=1}^{N}S_{i}\left(t\right)=\frac{N_{↑}\left(t\right)-N_{↓}\left(t\right)}{N}=2c\left(t\right)-1.$$

For the analytical treatment c(t) is usually more convenient, because within the mean-field approach it gives the probability that a randomly chosen agent, at any site, is positive [3,30]. However, in case of the Landau approach an order parameter *m* will be used.

### 3.1. Time Evolution

The *q*-voter model is based on the random sequential updating, which means that in an elementary update only one spin can change its state and thus one of three events is possible: the concentration of the positive opinion $c=N_{↑}/N$ increases or decreases by 1/N or remains constant with the respective probabilities:

(4) $$\begin{array}{ccc} \gamma^{+}\left(c\right) & = & Prob\left\{c\to c+\frac{1}{N}\right\}\\ \gamma^{-}\left(c\right) & = & Prob\left\{c\to c-\frac{1}{N}\right\}\\ \gamma^{0}\left(c\right) & = & Prob\left\{c\to c\right\}=1-\gamma^{+}\left(c\right)-\gamma^{-}\left(c\right). \end{array}$$

For our model on the infinite (N→∞) complete graph:

(5) $$\begin{array}{ccc} \gamma^{+}\left(c\right) & = & \left(1-p\right)\left(1-c\right)c^{q_{c}}+p{\left(1-c\right)}^{q_{a}+1},\\ \gamma^{-}\left(c\right) & = & \left(1-p\right)c{\left(1-c\right)}^{q_{c}}+pc^{q_{a}+1}. \end{array}$$

Using probabilities given by Equation (5) we can simulate trajectories of a random variable c(t), as done in [3,30]. However, we can also write the evolution equation of the corresponding expected value. For N→∞ we can safely assume, which is confirmed also by Monte Carlo simulations [3] that random variable c(t) localizes to the expected value:

(6) $$c\left(t+\Delta t\right)=c\left(t\right)+\frac{1}{N}\left[\gamma^{+}\left(c\right)-\gamma^{-}\left(c\right)\right],$$

where Δt=1/N, because one Monte Carlo step corresponds to *N* elementary updates, i.e., NΔt=1.

Therefore for N→∞ we obtain the following rate equation:

(7) $$\frac{\partial c}{\partial t}=\gamma^{+}\left(c\right)-\gamma^{-}\left(c\right)=\left(1-p\right)\left[\left(1-c\right)c^{q_{c}}-c{\left(1-c\right)}^{q_{c}}\right]+p\left[{\left(1-c\right)}^{q_{a}+1}-c^{q_{a}+1}\right]$$

**Figure 1:**
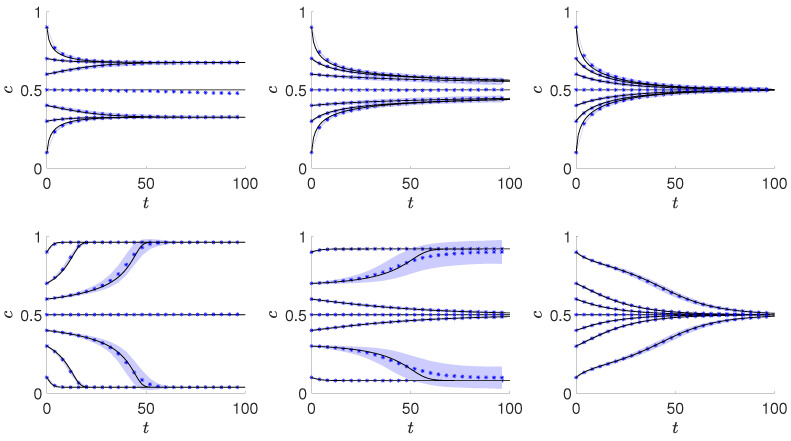
Average trajectories for $q_{a}=2$, two values of $q_{c}$ ($q_{c}=4$ in the upper and $q_{c}=8$ in the bottom panels) and several values of *p* increasing from left to right. Analytical results are marked by the solid lines, whereas Monte Carlo results are marked by symbols. Simulation results were obtained for the graph of size $N=10^{4}$ and averaged over 100 samples. The area between the bounded lines showing the standard deviation is marked by the light blue color. Exact values of *p* from left to right are the following: p=0.18,0.2,0.22 (upper panels, i.e., in for $q_{a}=2,q_{c}=4$) and p=0.03,0.05,0.07 (bottom panels, i.e., for $q_{a}=2,q_{c}=8$).

Sample trajectories obtained from Equation (6) and independently from the Monte Carlo simulations for the systems of size $N=10^{4}$ are shown in Figure 1. It is seen that the agreement between the Monte Carlo and the analytical results is high, even for relatively small system, what is expected in case of a complete graph. Therefore, all other results will be presented based on the analytical calculations.

Preliminary results, presented in Figure 1, suggest that there is a continuous phase transition for $q_{a}=2$ and $q_{c}=4$ (upper panels in Figure 1), whereas a discontinuous one for $q_{a}=2$ and $q_{c}=8$ (bottom panels in Figure 1). In the middle bottom panel metastability is visible, i.e., the final concentration of positive opinions depends on the initial state. In the metastable region a standard deviation is much larger for trajectories starting from c(0) near the borders of basins of attraction. To explore deeper the possibility of the existence of discontinuous phase transitions within the model, we calculate the dependence between the stationary value of $c=c_{st}$, as a function of the probability of anticonformity *p*.

### 3.2. Stationary States

To calculate stationary values of concentration $c=c_{st}$ we must solve the following equation:

(8) $$\frac{\partial c}{\partial t}=\gamma^{+}\left(c\right)-\gamma^{-}\left(c\right)=F\left(c\right)=0,$$

where F(c) plays a role of an effective force [30,39]. One solution of the above equation, namely c=1/2, is straightforward because it is seen that for c=1/2 the right side of quation (7) equals to zero. The stability of this solution can be checked within linear stability analysis [39]. The fixed point c=1/2 is stable if [39]:

(9) $${\left.\frac{\partial F(c)}{\partial c}\right|}_{c=\frac{1}{2}}={\left.\frac{\partial \gamma^{+}\left(c\right)}{\partial c}\right|}_{c=\frac{1}{2}}-{\left.\frac{\partial \gamma^{-}\left(c\right)}{\partial c}\right|}_{c=\frac{1}{2}}<0.$$

From Equation (5):

(10) $$\begin{array}{ccc} \frac{\partial \gamma^{+}\left(c\right)}{\partial c} & = & \left(1-p\right)\left[-c^{q_{c}}+q_{c}\left(1-c\right)c^{q_{c}-1}\right]-p\left(q_{a}+1\right)c^{q_{a}},\\ \frac{\partial \gamma^{-}\left(c\right)}{\partial c} & = & \left(1-p\right)\left[{\left(1-c\right)}^{q_{c}}-q_{c}c{\left(1-c\right)}^{q_{c}-1}\right]+p\left(q_{a}+1\right)c^{q_{a}}, \end{array}$$

and thus, we obtain:

(11) $${\left.\frac{\partial F(c)}{\partial c}\right|}_{c=\frac{1}{2}}=2\left[\left(1-p\right)\frac{q_{c}-1}{2^{q_{c}}}-p\frac{q_{a}+1}{2^{q_{a}}}\right]<0.$$

It means that c=1/2 is a stable fixed point for

(12) $$p>p_{1}^{*}\left(q_{a},q_{c}\right)=\frac{2^{q_{a}}\left(q_{c}-1\right)}{2^{q_{a}}\left(q_{c}-1\right)+2^{q_{c}}\left(q_{a}+1\right)}.$$

At $p=p_{1}^{*}\left(q_{a},q_{c}\right)$ the fixed point c=1/2 loses stability and for $p<p_{1}^{*}\left(q_{a},q_{c}\right)$ it becomes unstable, which is also visible in Figure 1.

Unfortunately, we are not able to calculate analytically other steady states, i.e., generally solve Equation (8) in the form $c_{st}=c_{st}\left(p\right)$ for arbitrary values of $q_{a}$ and $q_{c}$. However, following [30] we can easily derive the opposite relation:

(13) $$p=\frac{c_{st}{\left(1-c_{st}\right)}^{q_{c}}-\left(1-c_{st}\right)c_{st}^{q_{c}}}{c_{st}{\left(1-c_{st}\right)}^{q_{c}}-\left(1-c_{st}\right)c_{st}^{q_{c}}+{\left(1-c_{st}\right)}^{q_{a}+1}-c_{st}^{q_{a}+1}}$$

**Figure 2:**
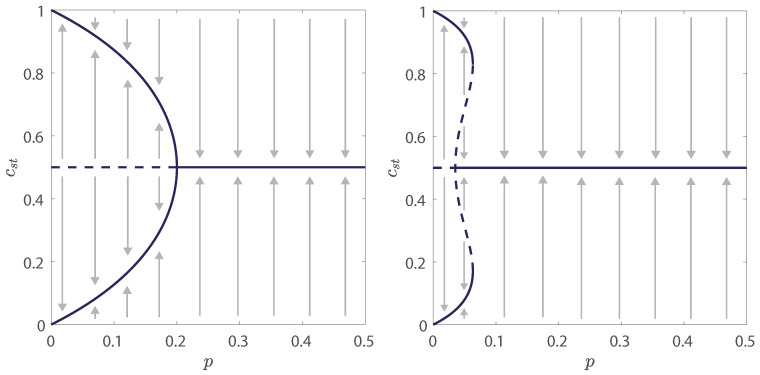
Flow diagrams for $q_{a}=2$ and two values of $q_{c}$: $q_{c}=4$ (left panel) and $q_{c}=8$ (right panel), which are the same values as in Figure 1. Here solid lines denote stable steady values of concentration $c_{st}$, whereas dashed lines denote unstable values of $c_{st}$. Arrows denote the direction of flow, i.e., how the concentration changes in time. Supercritical pitchfork bifurcation that corresponds to the continuous phase transition is seen in the left panel, whereas subcritical pitchfork bifurcation that corresponds to the discontinuous phase transition is seen in the right panel [28,39].

**Figure 3:**
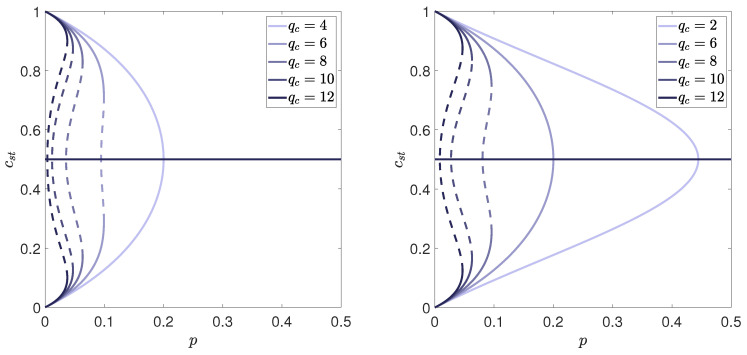
Dependence between the stationary concentration of positive opinions $c_{st}$ and the probability of anticonformity *p* for $q_{a}=2$ (**left panel**) and $q_{a}=4$ (**right panel**).

We use the above formula to plot $c_{st}=c_{st}\left(p\right)$ simply rotating the figure $p=p(c_{st})$. Results for $q_{a}=2$ and $q_{a}=4$ are presented in Figure 2 and Figure 3. Although Figure 2 shows only two examples ($q_{a}=2,q_{c}=4$ and $q_{a}=2,q_{c}=8$), out of many presented in Figure 3, it helps to better visualize what actually happens with the system. First of all, it is visible that there is a phase transition between phase with up-down (yes-no) symmetry and the phase in which this symmetry is broken, i.e., one of two opinions wins.

Moreover, it is seen that the phase transition is continuous for some values of parameters $q_{a}$ and $q_{c}$, whereas for others it is discontinuous. For example, for the fixed value $q_{a}=2$ the transition is continuous for $q_{c}=4$ but for $q_{c}\geq 6$ it is discontinuous, see Figure 3. Similarly, for the fixed value $q_{a}=4$ the phase transition is continuous for $q_{c}=2$ and $q_{c}=6$, whereas for $q_{c}\geq 8$ it is discontinuous. In case of a discontinuous phase transition, hysteresis is clearly seen, which means that the system can reach ordered or disordered state depending, on the initial conditions. The direction in which the system moves is indicated by the arrows in Figure 2.

In case of a continuous phase transition function $p=p(c_{st})$ has only one extremum—a maximum at $c_{st}=1/2$. The value $p^{*}=p\left(1/2\right)$ is the critical point, above which the stationary state has an up-down symmetry. It should be also noticed that in this case point $p^{*}$ coincides with the bifurcation point $p_{1}^{*}$, given by Equation (12), at which the fixed point c=1/2 changes stability. From the perspective of nonlinear dynamics, point $p_{1}^{*}$ is a point of the supercritical pitchfork bifurcation [28,39], see left panel in Figure 2.

On the other hand, in case of a discontinuous phase transition function $p=p(c_{st})$ has 3 extrema—a minimum at $c_{st}=1/2$ and two maxima located symmetrically with respect to the line $c_{st}=1/2$ at $c_{st}=c_{+}>1/2$ and $c_{st}=c_{-}<1/2$. The value $p_{1}^{*}=p\left(1/2\right)$ is called the lower spinodal and $p_{2}^{*}=p\left(c_{+}\right)=p\left(c_{-}\right)$ is called the upper spinodal. For $p<p_{1}^{*}$ the system reaches a phase in which one of two opinions wins ($c_{st}\neq 1/2$), independently of the initial state of the system. On the other hand, for $p>p_{2}^{*}$ the system reaches a phase with up-down symmetry ($c_{st}=1/2$), independently of the initial state of the system. The most interesting is the metastable region which corresponds to $p\in (p_{1}^{*},p_{2}^{*})$. In this region there is a phase coexistence and the stationary state depends on the initial one. In this case, the bifurcation point $p_{1}^{*}$, given by Equation (12) coincides with the lower spinodal. From the perspective of nonlinear dynamics, point $p_{1}^{*}$ is a point of the subcritical pitchfork bifurcation [28,39], see right panel in Figure 2.

To derive the phase diagram, we need to calculate:

1. **The lower spinodal as a function of parameters $q_{a},q_{c}$**. It corresponds to the value of $p=p_{1}^{*}$ at $c_{st}=1/2$, so it can be easily calculated from the relation $p=p(c_{st})$ given by Equation (13). In the case of a continuous phase transition this is simply the critical point $p^{*}=p_{1}^{*}$, which separates two phases. In this case, it corresponds to the maximum of $p=p(c_{st})$, whereas in case of a discontinuous phase transition it corresponds to the minimum of $p=p(c_{st})$, as seen in Figure 2 and Figure 3. As already written, $p=p_{1}^{*}$ is also a pitchfork bifurcation point, given by Equation (12), at which a steady state c=1/2 changes stability, i.e., for $p<p_{1}^{*}$ an agreement phase (c≠1/2) is stable and disagreement phase (c=1/2) is unstable. This indicates that independently on the initial state of the system an agreement phase is reached. Although $p_{1}^{*}$ has been already calculated within linear stability analysis, we will show that indeed it can be also obtained from Equation (13).
2. **The tricritical point, i.e., the value of $q_{c}=q_{c}^{*}$ as a function of $q_{a}$ for which the transition switches from continuous to discontinuous.** As described above at this point the minimum at $c_{st}=1/2$ changes to maximum and thus this point can be also easily derived by calculating the point in which the second derivative of *p* changes the sign.
3. **The upper spinodal $p=p_{2}^{*}$ as a function of parameters $q_{a},q_{c}$.** As written above, in case of discontinuous phase transition $p=p(c_{st})$ has two maxima at $c_{+}$ and $c_{-}$ and the value $p\left(c_{+}\right)=p\left(c_{-}\right)=p_{2}^{*}$ is the upper spinodal, so it can be also derived from the relation $p=p(c_{st})$ given by Equation (13). In theory calculations are straightforward. Unfortunately, it occurs that finding an analytical formula for $p_{2}^{*}=p_{2}^{*}\left(q_{a},q_{c}\right)$ for arbitrary values of parameters $q_{a}$ and $q_{c}$ is impossible and the upper spinodal will be obtained numerically.
4. **The point of the phase transition $p^{*}=p^{*}\left(q_{a},q_{c}\right)$.** For a continuous phase transition, it is straightforward, as described above, because it corresponds to the value of $p=p_{1}^{*}$ at $c_{st}=1/2$. In fact, for a continuous phase transition all three points: lower spinodal $p_{1}^{*}$, upper spinodal $p_{2}^{*}$ and the point of the phase transition $p^{*}$ collapse to the single critical point, i.e., $p^{*}=p_{1}^{*}=p_{2}^{*}$. For a discontinuous phase transition, it is far less trivial. The transition point is placed between lower and upper spinodals. In thermodynamics it corresponds to the point, at which phases are in the equilibrium, i.e., corresponding thermodynamic potential has minima of equal depth. Here we will also introduce an equivalent of a potential and use it to calculate the transition point.

As written above, the lower spinodal corresponds to the value of *p* at $c_{st}=1/2$. However, for this value denominator in Equation (13) equals zero and therefore we have to take the limit c→1/2 and use L‘Hospital’s rule:

(14) $$p_{1}^{*}\left(q_{a},q_{c}\right)=\lim_{c\to \frac{1}{2}}p\left(c,q_{a},q_{c}\right)\overset{H}{=}\frac{2^{q_{a}}\left(q_{c}-1\right)}{2^{q_{a}}\left(q_{c}-1\right)+2^{q_{c}}\left(q_{a}+1\right)}\overset{q_{a}=q_{c}=q}{\to }=\frac{q-1}{2q}$$

First of all, we see that indeed we obtained exactly the same result as within linear stability analysis, see Equation (12). Moreover, it is seen that for $q_{a}=q_{c}=q$ the results reduces to the formula for the original *q*-voter model with anticonformity, derived in [30]. On the other hand, if we put $q_{a}=1$ and $q_{c}=q$ we obtain:

(15) $$p_{1}^{*}\left(1,q\right)=\frac{2(q-1)}{2\left(q-1\right)+2^{q+1}}=\frac{q-1}{q-1+2^{q}},$$

whereas for the model with independence, introduced in [30] the following result was obtained:

(16) $$p_{1}^{*}\left(q\right)=\frac{q-1}{q-1+2^{q-1}},$$

which is very close to the result for the *q*-voter model with anticonformity for $q_{a}=1$ and $q_{c}=q$. So, it seems that changing $q_{a}$ for the fixed value of $q_{c}$ or vice versa we can tune the model from the ‘anticonformity’ regime to the ‘independence’ regime, in which discontinuous phase transitions are possible. However, it should be stressed here that this result may be valid only in case of a complete graph, i.e., when we neglect all spatial correlations. In this case, both types of nonconformity, anticonformity and independence, tries to disorder the system. However, in general anticonformity should support active bonds, whereas independence introduces just random changes and thus we expect that on graphs with high clustering coefficient the difference between two types of nonconformity would be stronger. To check this prediction we plan to investigate the model on different graphs.

As written above, we can easily calculate the tricritical value $q_{c}^{*}=q_{c}^{*}\left(q_{a}\right)$, for which transition changes from continuous to discontinuous, from the following condition:

(17) $$\lim_{c_{st}\to \frac{1}{2}}\frac{\partial^{2}p}{\partial c_{st}^{2}}=0\Leftrightarrow q_{c}=q_{c}^{*}=\frac{1}{2}\left(5+\sqrt{25-4q_{a}+4q_{a}^{2}}\right).$$

To calculate the upper spinodal we should first find points at which $p=p(c_{st})$ has maxima, but for this the following equation has be solved for $c_{st}$:

(18) $$\frac{\partial p}{\partial c_{st}}=0,$$

which is in general impossible within an analytical treatment. However, it can be easily done numerically.

Yet, it is less obvious how to calculate the point of the discontinuous phase transition. One natural possibility to solve the problem is to use the Landau approach, similarly as it was done in [30]. We have to stress here that in [30] only the lower spinodal was calculated within this approach. However, in general it could be used to derive analytical formulas also for the upper spinodal, the point of the phase transition, as well as the tricritical point. Before we proceed to apply the Landau approach, let us describe the method in an accessible way for researchers not trained in the theory of phase transitions.

### 3.3. Landau Approach for Continuous and Discontinuous Phase Transitions

Landau theory was originally proposed to describe continuous phase transitions [38]. Landau introduced an order parameter, here the stationary value of *m* given by Equation (3), to distinguish between phases of the system: m=0 in a disordered state (traditionally above the critical point), whereas m≠0 in an ordered phase (traditionally below the critical point). Additionally, Landau assumed that the thermodynamical potential, originally the Gibbs free energy, is not only a function of certain thermodynamical quantities, such as temperature and pressure, but it is also a function of an order parameter. In general, different potential could be used, for example a potential V=V(m,p) as a function of the order parameter *m* and the probability of nonconformity *p* has been introduced for the *q*-voter model in [30] and here we will use the same approach. Finally, Landau assumed that the value of *m* near the critical point is small and thus the potential can be expanded into a power series:

(19) $$\begin{array}{ccc} V & = & V_{0}+\frac{1}{1!}\frac{\partial V}{\partial m}m+\frac{1}{2!}\frac{\partial^{2}V}{\partial m^{2}}m^{2}+\frac{1}{3!}\frac{\partial^{3}V}{\partial m^{3}}m^{3}+\frac{1}{4!}\frac{\partial^{4}V}{\partial m^{4}}m^{4}+\ldots ,\\ & = & V_{0}+A_{0}m+Am^{2}+B_{0}m^{3}+Bm^{4}+\ldots . \end{array}$$

For a system with up-down symmetry, V(−m)=V(m), all odd terms must disappear and thus $A_{0}=B_{0}=\ldots =0$. If we additionally ignore higher powers of *m* we obtain:

(20) $$\begin{array}{ccc} V & = & V_{0}+Am^{2}+Bm^{4}. \end{array}$$

The condition of the stable equilibrium is equivalent to the condition for a minimum of the potential and thus:

(21) $$\begin{array}{c} \frac{\partial V}{\partial m}=2m\left(A+2Bm^{2}\right)=0,\;\frac{\partial^{2}V}{\partial m^{2}}=2\left(A+6Bm^{2}\right)>0. \end{array}$$

From the necessary condition for a minimum, i.e., the first condition of Equation (21), we obtain two solutions: m=0 (disordered phase) and $m^{2}=\pm -A/2B$ (ordered phase). Inserting these solutions into the condition of stability (sufficient condition for a minimum), i.e., the second condition of Equation (21), we obtain:

(22) $$\begin{array}{c} {\left.\frac{\partial^{2}V}{\partial m^{2}}\right|}_{m=0}=2A>0\Rightarrow A>0,\;{\left.\frac{\partial^{2}V}{\partial m^{2}}\right|}_{m^{2}=\pm -A/2B}=-4A>0\Rightarrow A<0, \end{array}$$

which means that *A* changes its sign during transition from one phase to another, i.e., the critical point corresponds to A=0. However, it should be noticed that the stability condition (21) at the critical point (i.e., for A=0) gives:

(23) $$\begin{array}{c} \frac{\partial^{2}V}{\partial m^{2}}=2\left(A+6Bm^{2}\right)=12Bm^{2}>0\Rightarrow B>0. \end{array}$$

**Figure 4:**
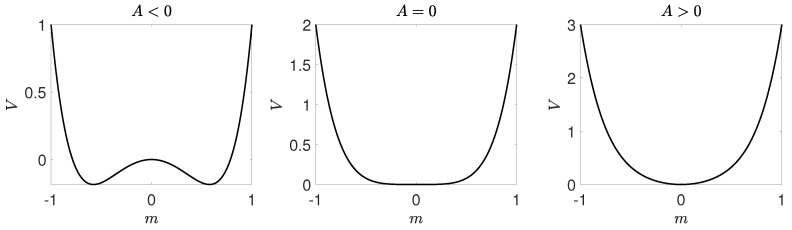
Potential given by Equation (20) for B>0(B=1) and three values of *A*: A<0(A=−1) (**left panel**), A=0 (**middle panel**) and A>0(A=1) (**right panel**).

Moreover, in order to obtain for A<0 real values of *m* corresponding to the ordered state, i.e., $m^{2}=\pm -A/2B$ we also need B>0. Therefore, **the Landau theory describes continuous phase transitions only for B>0**. In Figure 4 we present a potential, given by Equation (20), for B>0 (specifically for B=1) and three values of *A*: A=−1, A=0 and A=1. It is seen that indeed for A<0 the potential has three extrema: maximum at m=0 and two minima corresponding to $m^{2}=\pm -A/2B$. For A>0 the potential has only one extremum: minimum at m=0. This means that for A=0 the steady state m=0 loses stability. In the next section we will show that indeed this condition is equivalent with the condition given by Equation (12).

Although originally Landau theory was introduced for continuous phase transitions, it occurs that **for B<0,C>0 we can describe discontinuous phase transitions** [40]. At the end of this section we will see why the assumption C>0 is needed. For B<0 we have to take into account the next term of the power series:

(24) $$V=V_{0}+Am^{2}+Bm^{4}+Cm^{6}.$$

Again, we start with the condition for a stable equilibrium, i.e., minimum of the potential:

(25) $$\frac{\partial V}{\partial m}=2m\left(A+2Bm^{2}+3Cm^{4}\right)=0,\;\frac{\partial^{2}V}{\partial m^{2}}=2\left(A+6Bm^{2}+15Cm^{4}\right)>0$$

The necessary condition for a minimum, i.e., the first condition of Equation (25) is fulfilled if:

(26) $$m=0\;\mathrm{or}\;A+2Bm^{2}+3Cm^{4}=0.$$

**Figure 5:**
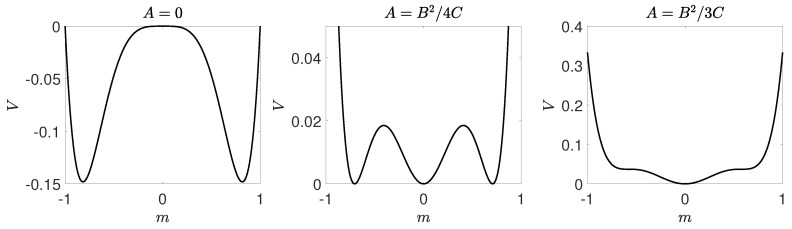
Potential given by Equation (24) for B<0(B=−1), C>0(C=1) and three values of *A*: A=0, which corresponds to lower spinodal (**left panel**), $A=B^{2}/4C$, which corresponds to the point of the phase transition (**middle panel**) and $A=B^{2}/3C$, which corresponds to upper spinodal (**right panel**).

Let us first check the stability of the first solution, which corresponds to the disordered phase. Inserting this solution into the condition of stability (sufficient condition for a minimum), i.e., the second condition of Equation (25), we obtain:

(27) $$\begin{array}{c} {\left.\frac{\partial^{2}V}{\partial m^{2}}\right|}_{m=0}=2A>0\Rightarrow A>0,\; \end{array}$$

which means that for A>0 the solution m=0 is stable, whereas for A<0 it becomes unstable, analogously as in the case of continuous phase transition (i.e., for B>0). However, the behavior of the potential is now very different, which is visible in Figure 5.

It occurs that for a certain range of A>0, additionally to m=0, there are two other stable solutions given by the second condition in Equation (26):

(28) $$\begin{array}{c} m^{2}=\frac{-B\pm \sqrt{B^{2}-3AC}}{3C}. \end{array}$$

Because *m* has to be a real number, for B<0 and C>0 we obtain:

(29) $$\begin{array}{c} m^{2}=\frac{-B+\sqrt{B^{2}-3AC}}{3C}, \end{array}$$

if the following condition is fulfilled:

(30) $$\begin{array}{c} B^{2}-3AC>0\to A<\frac{B^{2}}{3C}. \end{array}$$

It means that for $A\in (0,B^{2}/3C)$ two different types of phases (ordered and disordered) can coexist and region of metastability is limited by the spinodals:

(31) $$\begin{array}{c} A_{1}=0,\;A_{2}=B^{2}/3C. \end{array}$$

Until now we have not yet calculated the point of the phase transition. For B>0 the phase transition corresponds to the condition A=0, i.e., to the critical point, in which the solution m=0 losses stability: for A<0 the system always reaches one of two symmetrical ordered states (only these are stable) and for A>0 the system always reaches disordered phase. For B<0 the condition A=0 still corresponds to the critical point, in which the solution m=0 losses stability. However, for $A\in (0,B^{2}/3C)$ the system can reach one of three different states, two ordered and one disordered, depending on the initial conditions. In thermodynamics the point of the phase transitions is defined as the point in which all phases are in the equilibrium, which is equivalent to the condition that potential V(0) for the disordered phase is equal to the potential V(m) of the ordered phases, where:

(32) $$\begin{array}{c} V\left(0\right)=V_{0},\;V\left(m\right)=V_{0}+Am^{2}+Bm^{4}+Cm^{6}, \end{array}$$

which leads to:

(33) $$\begin{array}{c} m^{2}\left(A+Bm^{2}+Cm^{4}\right)=0. \end{array}$$

From the condition of equilibrium (25) and from equality of potentials (33) we obtain the set of equations:

(34) $$\begin{array}{c} \left.\left\{\begin{array}{c} A+2Bm^{2}+3Cm^{4}=0\\ A+Bm^{2}+Cm^{4}=0 \end{array}\right.\right|-\Rightarrow m^{2}\left(B+2Cm^{2}\right)=0\Rightarrow m=0,m^{2}=-\frac{B}{2C}. \end{array}$$

Now we see why for B<0 we had to assume that C>0. For C<0 solutions would be complex. Inserting solution $m^{2}=-B/2C$, which corresponds to the jump of the order parameter at the transition point, to the condition (33) we obtain that at the phase transition:

(35) $$\begin{array}{c} A=\frac{B^{2}}{4C}. \end{array}$$

In Figure 5 we see that indeed for this value of *A* the potential has three minima of equal depth.

### 3.4. Application of the Landau Approach

To use this approach, we have to define an equivalent of a potential, which we called an effective potential [30]:

(36) $$\begin{array}{ccc} V(c)=-\int F(c)dc & = & \frac{1-p}{\left(q_{c}+1\right)\left(q_{c}+2\right)}\left[c^{q_{c}+1}\left(c\left(q_{c}+1\right)-q_{c}-2\right)-{\left(1-c\right)}^{q_{c}+1}\left(c\left(q_{c}+1\right)+1\right)\right]\\ & + & \frac{p}{q_{a}+2}\left[c^{q_{a}+2}+{\left(1-c\right)}^{q_{a}+2}\right]. \end{array}$$

It is worth mentioning that potentials, defined as above, are used also in nonlinear dynamics as an alternative way to visualize the dynamics of the first-order system [39]. Within such an approach system always moves toward lower potential. Extrema of the potential (equilibrium states) correspond to fixed points: minima of *V* correspond to stable fixed points and maxima correspond to unstable fixed points.

To use the classical Landau approach, we need to express the above potential in terms of the order parameter given by Equation (3):

(37) $$\begin{array}{ccc} V(m) & = & \frac{1-p}{\left(q_{c}+1\right)\left(q_{c}+2\right)2^{q_{c}+2}}{[\left(1+m\right)}^{q_{c}+1}\left(\left(1+m\right)\left(q_{c}+1\right)-2q_{c}-4\right)\\ & - & {\left(1-m\right)}^{q_{c}+1}\left(\left(1+m\right)\left(q_{c}+1\right)+2\right)]\\ & + & \frac{p}{\left(q_{a}+2\right)2^{q_{a}+2}}\left[{\left(1+m\right)}^{q_{a}+2}+{\left(1-m\right)}^{q_{a}+2}\right]. \end{array}$$

Now we expand the above potential into power series and keep only the first three terms of the expansion:

(38) $$V\left(m\right)=Am^{2}+Bm^{4}+Cm^{6},$$

where:

(39) $$\begin{array}{ccc} A & = & \frac{1}{4}\left(\frac{p(q_{a}+1)}{2^{q_{a}}}-\frac{\left(1-p\right)\left(q_{c}-1\right)}{2^{q_{c}}}\right),\\ B & = & \frac{1}{48}\left(\frac{p\left(q_{a}-1\right)q_{a}\left(q_{a}+1\right)}{2^{q_{a}}}-\frac{\left(1-p\right)\left(q_{c}-5\right)\left(q_{c}-1\right)q_{c}}{2^{q_{c}}}\right),\\ C & = & \frac{1}{1440}\left(\frac{p\left(q_{a}-3\right)\left(q_{a}-2\right)\left(q_{a}-1\right)q_{a}\left(q_{a}+1\right)}{2^{q_{a}}}\right.\\ & - & \left.\frac{\left(1-p\right)\left(q_{c}-9\right)\left(q_{c}-3\right)\left(q_{c}-2\right)\left(q_{c}-1\right)q_{c}}{2^{q_{c}}}\right). \end{array}$$

**Figure 6:**
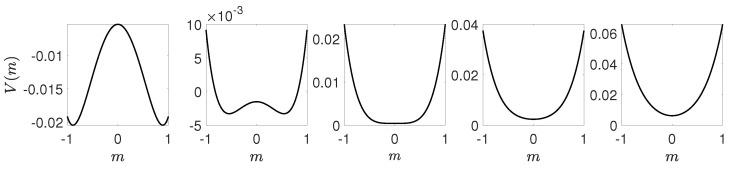
Potential for $q_{a}=2,q_{c}=4$ and several values of the probability of anticonformity. From left to right: $p<<p^{*},p<p^{*},p=p^{*},p>p^{*},p>>p^{*}$, where $p^{*}=0.2$ and exact values of *p* from left to right are the following: p=0.05,0.15,0.2,0.25,0.35.

**Figure 7:**
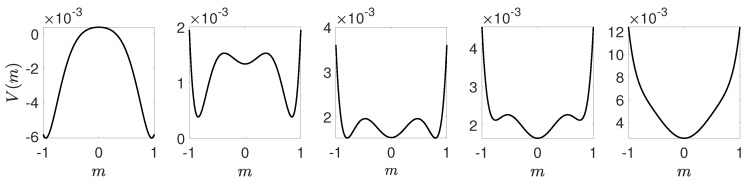
Potential for $q_{a}=2,q_{c}=8$ and several values of the probability of anticonformity. From left to right: $p<p_{1}^{*},p\in \left(p_{1}^{*},p^{*}\right),p=p^{*},p\in \left(p^{*},p_{2}^{*}\right),p>p^{*}$, where $p^{*}=0.0564$ and exact values of *p* from left to right are the following: p=0.02,0.05,0.0564,0.06,0.09.

We will use the results obtained within Landau approach, described in the previous section, which can be summarized as follows:

- The critical point $p=p_{1}^{*}$ at which solution m=0(c=1/2) loses stability corresponds to A=0.
- For B=0 there is a tricritical point at A=0, which means that for B>0 the transition is continuous, whereas for B<0 it is discontinuous.
- For B>0 the transition is continuous, see Figure 6. In such a case potential takes one of two forms. For $p<p_{1}^{*}$ potential *V* is a double-well one with maximum at m=0 (c=1/2). It means that the system always reaches one of two ordered phases: it is attracted by the minima of *V* and repelled by the maximum at m=0, which corresponds to the unstable fixed point. For $p>p_{1}^{*}$ the potential has only one minimum that corresponds to m=0 (c=1/2). It means that a system always reaches disordered phase, i.e., the fixed point m=0 (c=1/2) is stable.
- For B<0 the phase transition is discontinuous, see Figure 7, and we can calculate the transition point $p^{*}$ from the condition (35) as well as spinodals $p_{1}^{*},p_{2}^{*}$ from (31). As previously, the potential has two minima (ordered state) and one maximum (disordered state) for $p<p_{1}^{*}$, i.e., below lower spinodal. For $p\in (p_{1}^{*},p_{2}^{*})$ the potential has five extrema: two maxima corresponding to unstable fixed points and three minima corresponding to stable fixed points. Finally, for $p>p_{2}^{*}$ the potential has only one minimum that corresponds to m=0 (c=1/2).

We start with the critical point $p=p_{1}^{*}$ at which solution m=0(c=1/2) loses stability:

(40) $$A=0\Leftrightarrow p=p_{1}^{*}=\frac{2^{q_{a}}\left(q_{c}-1\right)}{2^{q_{a}}\left(q_{c}-1\right)+2^{q_{c}}\left(q_{a}+1\right)}.$$

We see that within the Landau approach we have obtained exactly the same value as previously in Equation (14). The transition is continuous as long as B>0 and for B<0 it is discontinuous, so we obtain the tricritical point from the condition:

(41) $$\begin{array}{cc} B(q_{a},\;q_{c},\;p_{1}^{*})=0 & \Leftrightarrow q_{c}=\frac{1}{2}\left(5+\sqrt{25-4q_{a}+4q_{a}^{2}}\right). \end{array}$$

Again, the Landau approach gives exactly the same value as obtained previously in Equation (17). So far, we did not obtain any new result, but we have just shown that two approaches give exactly the same results, which is expected in case of a complete graph. However, at least theoretically, the Landau approach allows also to calculate upper spinodal, as well as the transition point for discontinuous phase transition. The upper spinodal can be calculated from the condition $A=B^{2}/3C$, whereas the transition point from $A=B^{2}/4C$, as long as C>0. Unfortunately, there are two problems: (1) analytical solution is difficult due to the form of coefficients A,B and *C* and more importantly (2) *C* becomes negative shortly after the tricritical point is reached and thus we are able to obtain results only for 3–4 values of $q_{c}>q_{c}^{*}$.

Therefore, we obtain the transition point numerically from the original form of the potential given by Equation (37). In Figure 6 and Figure 7 potentials for $q_{a}=2,q_{c}=4$ and $q_{a}=2,q_{c}=8$ are shown respectively for several values of *p*. In Figure 6 we observe a typical behavior for a continuous phase transition, which mean that for $p<p^{*}$ there are two symmetrical equally deep minima at $m=m_{+}>0$ and $m=m_{-}<0$ corresponding to two ordered phases: positive or negative opinion wins. With increasing *p* minima are approaching each other and become shallower and finally for $p=p^{*}$ they collapse to a single minimum at m=0. On the other hand, in Figure 7 we observe a typical behavior for a discontinuous phase transition, which means that between the spinodals, i.e., for $p\in (p_{1}^{*},p_{2}^{2})$ there are 3 minima: two symmetrical $m_{-}$ and $m_{+}$ for the ordered phase and the third one at m=0 corresponding to the disordered phase. At the transition point all three minima are equally deep and this allows us to calculate $p^{*}$ for a discontinuous phase transition.

**Figure 8:**
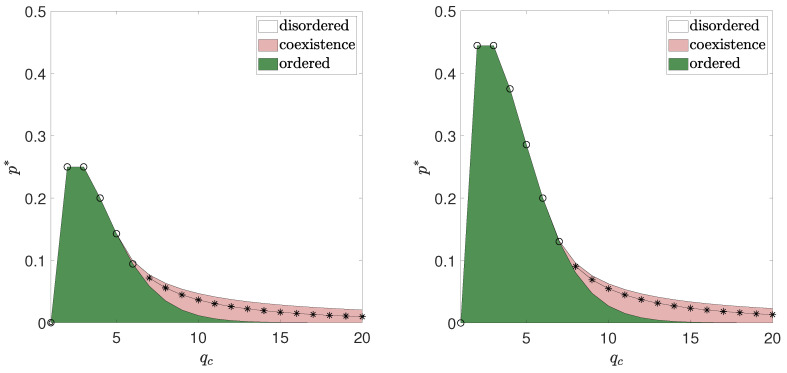
Phase diagrams for $q_{a}=2$ (**left panel**) and $q_{a}=4$ (**right panel**). Points of continuous phase transitions are marked by ∘ and discontinuous by ∗. Solid lines without symbols denote spinodal lines, i.e., limits of the region with metastability, in which the final state depends on the initial one.

Collecting all results obtained above, we can draw the phase diagram. Examples of phase diagrams for two values of $q_{a}$ are shown in Figure 8. These diagrams remind the one for the *q*-voter model with independence, presented in [30]. In both cases the maximum value is obtained for $p^{*}\left(q_{c}=2\right)=p^{*}\left(q_{c}=3\right)$, which means that agreement is more likely when the size of the group needed for conformity is of size 2 or 3 and then decreases. Moreover, for $q_{c}>q_{c}^{*}\left(q_{a}\right)$ the transition becomes discontinuous. It means that hysteresis appears, i.e., there is an interval of width $p_{2}^{*}-p_{1}^{*}$ between lower and upper spinodal in which the final state depends on the initial one and in which both phases (agreement and disagreement) coexist with each other. In this interval both phases are metastable: for $p<p^{*}$ the agreement is more stable (represented by deeper minimum of the potential), whereas for $p>p^{*}$ the disagreement is more stable.

**Figure 9:**
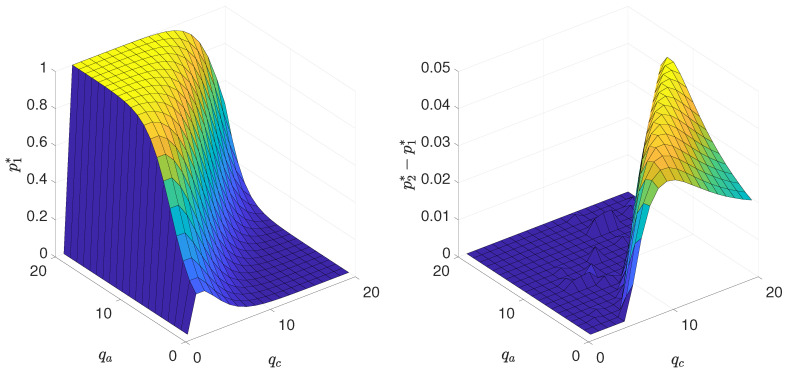
**Left panel**: the critical point $p_{1}^{*}$, below which the system is ordered (m≠0) independently on the initial state, as a function of $q_{a}$ and $q_{c}$. **Right panel**: the width of hysteresis $p_{2}^{*}-p_{1}^{*}$ as a function of $q_{a}$ and $q_{c}$.

To illustrate results for all values of $q_{a}$ and $q_{c}$ we have decided to plot the lower spinodal line $p_{1}^{*}$ (left panel in Figure 9), as well as the width of hysteresis, which is defined as a distance between spinodals, (right panel in Figure 9) as a function of both parameters $q_{a}$ and $q_{c}$. In the left panel in Figure 9 it is visible that for the fixed value of $q_{a}$ the dependence between $p^{*}$ and $q_{c}$ is non-monotonic and for all $q_{a}$ there is a maximum for $p^{*}\left(q_{c}=2\right)=p^{*}\left(q_{c}=3\right)$, which reminds the results for the *q*-voter model with independence [30]. On the other hand, for the fixed value of $q_{c}$ the transition point $p^{*}$ increases monotonically with $q_{a}$, which reminds the results for the *q*-voter model with anticonformity [30]. The width of hysteresis (right panel in Figure 9) is increasing rapidly when the tricritical point is crossed, then reaches certain maximal value and then decreases again, which means that there are ‘optimal’ values of $q_{a}$ and $q_{c}$ for which the interval of metastability is the largest.

## 4. Conclusions

In this paper, we proposed a generalized version of the *q*-voter model with anticonformity [30]. The generalization was based on the observation that there are no indications, neither from the social experiments nor from the social observations, to justify the assumption that the size of the group of influence inducing conformity is the same as the size of the group effecting in anticonformity. For example, within laboratory social experiments, it was repeatedly shown what there is a strong impact of the size of the influence group and its unanimity on conformity, for a review check [37]. For instance, it has been shown that it is essential for the group of influence to be unanimous and of sufficient size in order to persuade others. On the other hand, anticonformity is much less investigated. One of the reasons is the difficulty in designing the appropriate experiment. One of the few experiments on anticonformity was conducted by [41] but within this experiment the impact of the size of the group of influence has not been examined. Due to our knowledge there is no other laboratory experiment that checked the role of the size of the influence group on the anticonformity.

Therefore, we decided here to abandon the assumption made in [30] and check if this would change the type of the phase transition. It has occurred that indeed phase transition switches to a discontinuous one if only the size of the group of influence needed for conformity $q_{c}$ is sufficiently larger than the size of the group of influence needed for anticonformity $q_{a}$. We were able to find the analytical formula $q_{c}^{*}=q_{c}^{*}\left(q_{a}\right)$ for the tricritical point above which the transition becomes discontinuous, as well as the analytical formula for the critical point $p_{1}^{*}=p_{1}^{*}\left(q_{a},q_{c}\right)$, below which an up-down symmetry is broken, within two independent methods. Results obtained here reduces to those obtained in [30] for $q_{a}=q_{c}=q$. Interestingly, under the assumption that $q_{a}$ and $q_{c}$ are integer numbers the formula (17) gives the simple relation that the transition is discontinuous if $q_{c}\geq q_{a}+3$ for any $q_{a}>3$. For smaller values of $q_{a}\leq 3$ the formula (17) gives the condition $q_{c}\geq q_{a}+4$.

Results obtained here has encouraged us to design a laboratory experiment, based on the idea introduced in [41], to check how the size of the influence group impacts the level of anticonformity. However, it is too soon to provide more details of this project, as reported in [14].

In this paper, we investigated the model exclusively on a complete graph to make a comparison with the original *q*-voter model with anticonformity. However, it would be desired task for the future to study the model on various complex networks, because till now the *q*-voter model with anticonformity was studied only on the complete graph, even in its original formulation.
